# The Oculist's Vade-Mecum: A Complete Practical System of Ophthalmic Surgery

**Published:** 1844-04-01

**Authors:** 


THE OCULIST'S VADE-MECUM: A COMPLETE PRACTICAL SYSTEM OF OPHTHALMIC SURGERY. With numerous Wood-cuts and Coloured Engravings of the Diseases and Operations on the Eye. By *John Walker*, Surgeon to the Manchester Eye Hospital, &c. London, Longman and Co. Pp. 400. 12mo.

THE volume before us is intended to supply the profession with a clear, condensed, practical account of the present state of ophthalmic pathology and practice, especially adapted to the wants of the student and busy practitioner. Mr. Walker is already favourably known to the profession as a diligent cultivator of this interesting department of medicine—having published a valuable course of lectures in the "Lancet," and some interesting works and essays at different periods. He is, therefore, well qualified for the task he has undertaken.

We find, on comparison, that the Oculist's Vade-mecum is composed of the lectures already mentioned; with some slight additions and alterations. We will allow Mr. Walker to explain his own reasons for collecting and publishing them in their present form.

"Notwithstanding," he remarks, "the existence of a considerable number of books on Diseases of the Eye, there still appears room for one whose special object is to afford *practical* information to the student, the young practitioner, and him whose active engagements render impossible the study of the more voluminous publications with which this branch of medical science has been enriched—the works of Mackenzie, Guthrie, Middlemore, Lawrence, Wardrop, and others—not to mention a long list of German and other foreign names, who have done much for its advancement. To give to the work that practical character, the account of the various diseases has been illustrated by a selection of some of the most important cases, which an extensive experience, acquired during twenty years of observation and practice in this branch of surgery, has afforded. It would have been easy to have enlarged the scope of the present publication: the difficulty consisted in making a judicious selection of such points as are most likely to interest the busy practitioner, and furnish him, in a somewhat condensed form, with that useful information, which he requires. The pictorial illustrations, explanatory of the more important diseases, and of the principal operations, will, it is hoped, add materially to the value of the work; and, with the written descriptions, constitute a faithful representation and record of the present state of Ophthalmic Surgery."—*Preface.*

There can be no doubt that a book of this description will prove a valuable boon to the profession—especially the student and busy practitioner—but we cannot agree with our author that it will be equally valuable to the junior practitioner. The commencement of practice furnishes, in general, abundance of time and opportunity for the perusal and digestion of more minute and voluminous treatises, and, above all, the more able and practical Monographs, Essays, and Dissertations—which often constitute the most valuable parts of medical literature.

A complete analysis of such a volume as the present, is, of course, out of the question; we shall content ourselves, by extracting such portions as are either most interesting, original, or practical. Our author, after stating that, "in chronic ophthalmia, the conjunctival vessels appear to be distended and relaxed, and not to be possessed of their natural amount of tonic power," remarks that " the object to be kept in view, in treating a case of this description, is to restore the weakened vessels from their relaxed and enlarged condition, to their normal tone and calibre. How is this object best accomplished? Assuredly not by the use of leeches, blisters, and purgatives."—p. 14. These remarks appear to us somewhat prejudiced and one-sided, and the most successful practitioners will we think concur with us that our treatment can only be determined by the features of each individual case. In the words of Dr. Hocken (Lancet Vol. ii,—1842-3, p. 687), " we can only judge what is the best plan of treatment by attending to the actual conditions of the case—the history, and the general and local symptoms. We may meet with two cases of ophthalmia, of equal duration, but we must not on that account pursue a routine course for ' chronic' inflammation; for one case may present passive features, with a deranged and depressed condition of the general health, whilst the other may be active, with inflammatory fever. A routine practitioner does not trouble himself about these differences—he either depletes or stimulates both; one is cured whilst the other is rendered inveterate under his hands."

In the more severe and protracted cases the author recommends the application of either the nitrate of silver or the sulphate of copper in substance to the conjunctival surface of the lower lid.

" *Nitrate of Silver.*—The application of the nitrate of silver in substance is easily made, and is by far the most efficacious remedy I am acquainted with, for chronic ophthalmia. Expose the conjunctival surface of the inferior eyelid, by manipulating as before directed, and then draw the nitrate of silver, pointed like a pencil, *lightly* across it. The portion of conjunctiva touched, immediately becomes white, from the tears acting upon the nitrate of silver and producing, it is said, a muriate of silver. The application is always productive of a great increase in the lachrymal discharge, and is very generally followed by a severe smarting or burning sensation, which usually continues from *half an hour to three or four hours.* At the expiration of that period the uneasiness subsides, and a decided improvement is soon perceptible in the condition of the eye."

" *Sulphate of Copper.*—The application of the sulphate of copper in substance is also frequently productive of beneficial results; and, although much milder in its action than nitrate of silver, this remedy will generally be sufficiently powerful in the slighter cases of chronic conjunctivitis. It is to be applied in the same manner as the nitrate of silver; with this difference, that it should be kept in contact with the conjunctival membrane *for a few seconds,* which the patient will usually bear without much complaining. A small portion of the sulphate appears to be dissolved by the lachrymal fluid, as this fluid is generally perceived to be tinged of a blue colour, after the use of this substance." 15.

If compelled to employ these powerful remedies directly to the delicate surface of the conjunctiva, we should certainly prefer them in substance to strong solutions, as, according to our experience, they act more beneficially, and, if persisted in, are less likely to occasion a granular change. We are compelled, however, to enter our protest against the practice altogether—except where there is a tolerably free secretion of mucus or pus from the membrane—first, because it invariably occasions severe pain and distress, which lasts for a considerable time—from half an hour to three or four hours;—secondly, because it is quite unnecessary—the disease being more quickly, safely, and pleasantly cured by other means, quite independent of the excessive bleeding, purging, and mercurialization reprobated by the author; and thirdly, because if the remedy be persisted in, and used without excessive caution and matured judgment, it is very apt to occasion a disease much severer and more intractable than the primary complaint, viz. granular conjunctiva. We offer these opinions not as theories but as facts, having ourselves commenced practice, strongly prejudiced in favour of the nitrate of silver, and it was not until after witnessing repeatedly the agony it almost invariably occasioned,—the mean duration of which was certainly from two to three hours,—and the numerous cases of augmented inflammation, and granular disease which resulted from frequent and indiscriminate use, that we opened our eyes to what we are now fully convinced is the truth, viz. that it is applicable only to those diseases which are attended with a pretty free discharge of muco pus.

" *Ulceration from Nitrate of Silver.*—" There is one effect," says our author, " which I have sometimes observed to result from the use of nitrate of silver, which it is proper to mention before dismissing the subject of the local treatment of chronic ophthalmia. I mean superficial ulceration of the conjunctiva. In the vast majority of cases, this ulceration is followed by no disagreeable consequences—it is a matter of no importance; indeed, in some instances, it is probable that it may be rather beneficial than the contrary. Yet it occasionally happens that slight inversion of the lid results from it."

As far as we have seen, all the beneficial, without any of the evil effects of the nitrate, are obtained by applying the remedy to the outside of the lids instead of to the conjunctival lining. On this subject the author remarks.

" Mr. Higginbottom, the author of a valuable work on the use of nitrate of silver, recommends the application of this substance to the outer surface of the palpebræ. This mode of applying nitrate of silver has also recently been advocated by Mr. Wormald and Dr. Hocken, in the treatment of some forms of ophthalmia. This would be very proper if we could not obtain access to their conjunctival surface; but as this can always be accomplished without difficulty, it is obviously the part to which the remedy is to be applied."

The author has displayed not a little prejudice and want of correct information in the foregoing passage. We have carefully looked through the excellent little volume of Mr. Higginbottom, and feel persuaded that even the *idea* of using the remedy externally for the *cure of diseases of the eye* had never even suggested itself to his mind, much less had he put the plan in practice. He mentions, indeed, having used the remedy in erysipelas of the face " near the right eye," and in another case of the same disease he says, " I applied the nitrate of silver, as in the former case, upon and beyond the inflamed surface, except on the eyelids; having found it in former cases, when applied to that part, to occasion a considerable flow of tears, which, passing over the escharred surface, is rather troublesome, inducing excoriation." Again, at p. 175, he mentions a case of " irritable ulceration *near the eye* which was much benefited by this treatment." It is obvious, however, that ulceration near the eye, or erysipelas of the face have nothing to do with our present points of consideration. So much for want of correct information; our second charge is that of prejudice.

It is quite clear that the author has never even given the external application of the nitrate a trial, much less has he instituted an unprejudiced comparison between the benefits to be obtained by its external and internal use—its application to the integument of the eyelids, and to the surface of the conjunctiva—and carefully weighed the disadvantages which may attend either. Even allowing that both modes of use were equally beneficial, it is obvious that the external application is a much more simple operation and one more easily performed; but when it is considered that very slight and transient irritation attends this plan, and severe and protracted agony its application to the conjunctiva, and that the former *never* occasions any injurious consequences, whilst the latter, without great care and judgment, *often* does, that person must be prejudiced indeed who refuses to try a plan so simple, and safe, and the beneficial effects of which rest on such good authority, and extensive experience. Starting ourselves with a prejudice in favour of the application of the nitrate of silver to the conjunctiva itself, we have carefully weighed the advantages and disadvantages of its external and internal use, and unequivocally declare our firm conviction of the decided superiority of the former plan.

The author's objection to this is, moreover, not very logical. "This," he remarks, "would be very proper if we could not obtain access to the conjunctival surface, but as this can always be accomplished without difficulty, it is obviously the part to which the remedy is to be applied."

Q. Why is the conjunctiva the part to which the remedy ought to be applied? A. Because it can always be done without difficulty! But, notwithstanding this specious reasoning, we think the external use very proper, notwithstanding the easy—often too easy—access to the conjunctival surface.

Our author gives some good advice on the—

### *Mode of applying Local Remedies.*

"It is," he remarks, "rarely necessary to apply stimulants to the conjunctiva of the superior eyelid; because in simple conjunctival inflammation we seldom find that it participates to any considerable extent in the general inflammatory condition of the membrane (?); and it is the less necessary as the effect produced by their application to the lower one becomes diffused over the whole conjunctival surface by the winking motions. Indeed, the conjunctiva of the lower lid ought to be the recipient of all the local stimulants employed in chronic ophthalmia. If we prescribe a stimulating lotion or ointment, but little good can be expected to result from its use if this be not brought into actual contact with it; and, as we know how seldom applications of this kind are properly used by patients, there is the greater necessity for the surgeon himself frequently to apply something on which he can depend for producing the proper impression. If the application of stimulant fluids be entrusted to patients, or their attendants, strict injunctions should be given as to their efficient use. The lower eyelid ought to be depressed and everted, and a camel-hair pencil saturated with the fluid, should then be drawn across its conjunctival surface. If an ointment be recommended it should be first melted, and then applied in the same manner. In milder cases, the fluid may be dropped upon the conjunctiva oculi, or the ointment smeared upon the tarsal margins, but neither of these is so effective a means as the former." 16.

### Congenital Ophthalmia.

"This extraordinary case I will state a little in detail, since, as far as I know, there is no similar one on record, although, probably, others must have occasionally occurred.

"*Case.*—Mrs. T.'s child, when first brought under my notice, was six months old, and the mother, a very intelligent person, informed me that, at the time of birth, his eyes exhibited the same appearances as were now observable. The disease had run through its entire course previously to birth, for, according to her account, there was no puriform discharge, inflammation, or intolerance of light, noticed at any time subsequently. The cornea of one eye had completely sloughed, the eyeball had sunk, and, of course, not the slightest vision existed. More than one-half of the cornea of the other eye was opaque; through the remaining transparent portion a part of the pupil could be discerned, and the iris and cornea appeared almost in contact. The transparency gradually extended, and more of the pupil became accessible to light: thence, though vision was very imperfect, when I last saw the child, yet it appeared to be gradually improving."

The phenomena of this case are certainly peculiar, and seem to warrant the author in stating that, "after duly considering how perfectly the phenomena presented by the eyes of this child agree with those met with as results of purulent ophthalmia, attacking infants after birth, I think that no reasonable doubt can be entertained that they were occasioned by purulent ophthalmia which occurred before birth."

### Strumous Ophthalmia.

We fully agree with our author that the morbid sensibility to light, so strikingly manifested in this disease, is most erroneously attributed to a sympathetic affection of the retina. The same train of symptoms which characterise this disease, are temporarily produced by the intrusion of a particle of dust underneath the upper lid; in which case, it is obvious that all the symptoms must depend entirely on irritation of the conjunctival filaments of the ophthalmic division of the fifth pair of nerves.

"My own opinion," says our author, "is that sensibility to light, whether healthy or morbid, is to be referred to the fifth pair of nerves rather than to the retina. The retina I regard as simply the recipient of visual impressions. This is a subject which it would be obviously improper to enter upon here in detail; but I may relate a case which is interesting, in a practical point of view, and will serve to illustrate the distinction I have endeavoured to establish." 72.

In this case all the parts supplied by the fifth pair of nerves were paralysed on one side of the face. Taste was much impaired, but the eyes, with the exception of an inability to close the right eyelids, were perfectly healthy in appearance, and vision was perfect in both eyes. The author placed this patient before a very brilliant flame from a gas-burner. "The effect was very striking, for the left eye could not bear for a single instant the exposure to so vivid a light, whilst the opposite eye (that of the paralysed side), when placed close to it, was *completely insensible to its irritating influence*. He was treated on the combined antiphlogistic and mercurial plan, and speedily recovered. It was interesting to observe that, as common sensibility was restored, the eye became again susceptible of the irritating influence of light."

It was suggested by Dr. Hocken, in an Essay in the Lancet, (vol. i. 1842-3, p. 285,) that the retina itself is in direct communication with the fifth, through the medium of filaments, distributed with those of the optic nerve—not as Magendie thought, the actual nerve of vision, but the source of common sensibility to light, and the medium of impressions on the pupil, through the lenticular ganglion. The experiments of Fontana prove that the mere action of light on the iris is insufficient to produce contractions of the pupil unless the retina be simultaneously affected, whilst daily experience proves that the pupil contracts to a fine point from the mere influence of mechanical irritation of the conjunctiva. Dr. Hocken combining these facts with our author's interesting case just alluded to—of insensibility to light of the eye of the paralysed side whilst vision was perfect—some of Magendie's experiments, and, on the contrary, those cases of complete amaurosis, where the iris moves actively and freely, and where the patients can tell by their sensations the difference between light and darkness, without being able to see at all, remarks that—" These cases appear to dissect, as it were, the different portions of the retina, and show its separate functions; (in the latter,) the true retina is paralysed by disease in some part of the visual nervous system, whilst the sensitive functions of the fifth, and the motor of the third nerves remain intact," and in the former vice versâ.

### PTERYGIUM.

Our author agrees with Mr. Guthrie that Scarpa's hypothesis—that the resistance to the progress of pterygium upon the cornea is owing to the increasing adhesion between the conjunctival covering and the cornea itself, and that, on its arriving at the centre, the mechanical resistance is so excessive that it cannot be overcome, and that thus the further progress of a pterygium is stopped—" although it may appear sufficiently plausible, is so unsatisfactory, that the problem is still open for the exercise of further ingenuity."

The author's suggested explanation is that the conjunctiva receives blood from four distinct sources, each of which separately supplies one-fourth of the membrane, to a certain extent, independently of each other. These vessels pass into the substance of the conjunctiva at four distinct points—viz. one at each angle, and at the superior and inferior portions of the globe. In pterygium, these vessels, at whichever of the four sides the disease happens to be developed, proceed from that portion of the periphery of the conjunctival surface of the globe, and pass on towards, and upon the cornea to its centre, beyond which they never extend; thus occupying and being limited to one-fourth of the conjunctival surface, and therefore imparting to the diseased structure a triangular shape.

How would the author, however, explain such cases as Mr. Tyrrell states that he has seen, where the triangle was reversed—the base being situated on the cornea, and the apex at the periphery? The subject is of little importance in a practical point of view.

### Opacities of the Cornea.

The first object in the treatment of opacity is to remove any remaining inflammation. "The process by which an opaque deposit of any kind is removed from the texture of the cornea is that of absorption; and the only mode in which the surgeon can be of use, is in the employment of such remedies as have a tendency to increase the action of the vessels engaged in that process." After depositions of this kind, the absorbents are tardy in removing the superfluous matter—hence it is necessary to apply an artificial stimulus which shall excite the dormant energies of the vessels.

*A solution of the nitrate of silver* is the remedy most frequently used. Its strength should not exceed, in the first instance, two grains of the salt to an ounce of distilled water. "After a time, the proportion of the nitrate may be increased gradually up to ten grains to the ounce of water." In prescribing it, we must not forget that, when long-continued, it is apt to leave a permanent stain of a deep olive colour in the conjunctiva, which, in the case of young persons, and more particularly females, would constitute a serious blemish.

*Vinum opii* was formerly a favourite remedy. It would probably answer fully as well in the majority of cases.

*A solution of the bichloride of mercury* "is perhaps as good an application as any other. One grain of the salt to an ounce of distilled water is sufficiently strong to commence with; after a time, two or three grains to the ounce will be borne, but its strength ought to be increased very gradually, as it is considerably more irritating than the solution of nitrate of silver in like proportions. On the whole, it is decidedly preferable to the latter remedy, inasmuch as it never leaves any stain on the conjunctiva, however long its use may be continued."

Various stimulants are employed also in conjunction with unctuous matters. Of these, perhaps, the ung. hydrarg., nit. oxyd., the citrine, the ointment of nitrate of silver, and of the hydriodate of potass, are the best: they should be brought in contact with the conjunctival surface of the inferior lid, in the manner before pointed out.

An old-fashioned remedy, and our author thinks by no means the least effective, is that of blowing through a reed, or quill, some finely-powdered substance upon the surface of the eye, which would mechanically stimulate the absorbents.

In reviewing these remedies, we should certainly abstain from the use of a ten-grain solution of the nitrate of silver, as perfectly unnecessary, as well as for the reasons already given. We have found it necessary to select the remedy, and the strength at which it is most beneficial, not from any preconceived views, but from the actual conditions of the case we are about to treat, and from what we can gather by actual trial.

Moreover, remedies require to be occasionally alternated as well as cautiously increased in strength, as they seem to lose the beneficial influence, which they exerted in the first instance, by constant repetition.

> "It is only by the steady use of these remedies, perhaps for a considerable time, that any material improvement can be expected to result. Indeed I am inclined to think that they do not produce so much effect as is commonly attributed to them, for in many cases we find that the opacity is removed by the natural efforts, and in others, notwithstanding their use, it remains uninfluenced."

The absurd operations of removing the opaque portions of cornea, and bringing the edges of the wound together with sutures, paring off the opaque laminæ, and transplanting the tunic on the principle of the Taliacotian operation, are justly ridiculed by the author.

> "The only condition which offers a prospect of success by operation is in cases of central opacity where the pupil is obscured, and vision consequently destroyed. In such cases *an artificial pupil* may be formed opposite some portion of the transparent margin of the cornea, so as to give to the patient useful sight."

The author has omitted to mention the great benefit of belladonna in some of these cases. Wherever the pupil can be dilated to a sufficient extent to allow the rays of light to enter the interior of the eye beyond the margins of the opacity, all other treatment is unnecessary ; and hence it is applicable to a very large proportion of central opacities of the cornea.

"A particular kind of opacity of the cornea," says the author, "has been observed to follow the use of collyria containing salts of lead." We have no hesitation in stating that this is a mere chimera, as the opacity in question never results but from a peculiar chronic ulcer, which occurs in middle-aged individuals, and presents the same phenomena, whether local applications of all kind be religiously abstained from, or not ; as has been abundantly determined by the most able observers, over and over again.

### OPERATION FOR ARTIFICIAL PUPIL.

What circumstances are to be attended to before we shall be justified in resorting to an operation for artificial pupil?

1. That the eye has completely recovered from the effects of the morbid action, with the exception of the contracted state of the pupil, &c.

2. That the state of vision be such as to justify the operation ; for it is obvious that if vision remain sufficient to enable the patient to find his way about comfortably, or to read large type with optical assistance, it would be improper to perform an operation, which might (from the accidents which sometimes result from the most skilful proceedings), so far from being of service, leave him even worse than before. If, on the other hand, the vision of both eyes has been seriously damaged by the disease, or one has so suffered, whilst the other has been injured in some other way—leaving him unable to move about without a guide, then we shall be warranted in attempting some operative means for his improvement, since, if the attempt failed, his condition could scarcely be made worse.

An operation would be improper if the eye itself were amaurotic, independently of those causes which excluded the light from the interior of the organ. In these cases inflammation has extended to the retina and internal tunics. It would also be improper if a sufficiently large portion of the cornea did not retain its transparency.

It is sometimes a difficult matter to decide on the sensibility of the retina, because where the pupil is obliterated, and the capsule exceedingly opaque, an almost impenetrable barrier is offered to the passage of light into the interior of the eye. In this condition we must form our opinion from the general appearance of the eye—its vascularity, condition of the sclerotica, firmness or softness, enlargement or atrophy of the organ—and act accordingly. Moreover, if we find the anterior chamber obliterated, the iris in contact with the cornea, and more especially if the structure of the iris have been much changed, as denoted by its puckered and irregular appearance, it would imply the existence of so much mischief in the interior of the organ as to render the prognosis extremely doubtful.

If vision be obscured by opacity of the cornea, with or without closed pupil, it is necessary to consider if a sufficient portion of that texture retain its transparency, to allow of the transmission of light through an aperture to be formed in the iris beneath. When a moderate portion of the cornea remains perfectly transparent, or when the entire tunic is opaque, we have less difficulty in forming a prognosis; the doubtful cases being where there is a general opacity with only a partial and imperfect transparency at one or more points, and these of inconsiderable extent.

### Amaurosis.

Our author has considered the complex and difficult subject of nervous imperfection and loss of vision—viz. amaurosis and amaurotic affections—in two chapters, separated from one another by an intermediate chapter on the diseases of the humours. The first chapter is headed "Diseases of the Retina," and includes retinitis, or active amaurosis, amaurosis from chronic retinitis, sympathetic amaurosis, amaurosis from affections of the fifth nerve, amaurosis from general debility, hæmiopia, amaurotic cat's eye, glaucoma, muscæ volitantes, and injuries of the retina. The second chapter includes, under the denomination of "Supplementary Affections of the Eye," hemeralopia, nyctalopia, asthenopia, or weakness of vision, imperfect perception of colour, myopia, presbyopia, strabismus, and paralysis of the muscles of the eyeball. This arrangement is not very judicious.

The study is surrounded by difficulties on every side, partly from the impossibility of obtaining opportunities of examining the diseased tissues, during, or shortly after the existence of those pathological states, which give rise to loss of vision, and also in the diversity and obscurity of mere functional disturbances, which undoubtedly constitute the great majority of the transient and curable forms of the disease. Nevertheless it is full of interest, and well deserves the most careful consideration and diligent research. Many people, unwilling to devote that time and trouble to the subject which any difficult subject requires, think that it is unnecessarily complicated, resolutely shut their eyes against the truth, and loudly applaud any one who describes the disease, not as it exists in nature, but in their own indolent and ill-informed minds.

Our author has, on the whole, given a tolerably full and clear description of amaurosis (considering the scope of the work), although he has omitted much that might have been introduced, and has not even alluded to the congestive or hyperæmial forms which are comparatively common. Throughout it is apparent that the subject is one which he has studied without that zeal and energy conspicuous in some other parts of the volume, and to which he has not applied—probably from dislike—the full scope of his powerful mind. To quote his own words, " impairment of vision presents itself in such a protean variety of forms, and is described in such an endless diversity of terms, as to render its proper study a somewhat tedious and repulsive task."

*Acute Retinitis.*—The symptoms which characterise the commencement of this rare inflammation are "*sudden impairment* or even *complete loss of vision,* without any adequate affection of the iris, or of the adjacent transparent textures," combined with a disordered functional condition of the retina, " indicated by certain illusory appearances within or before the eye, such as *corruscations* or *flashes of light, coloured representations* of a red, sparkling, and variegated description, and, not unfrequently, *black moats* or clouds floating about."

A patient, whom we saw with this disease shortly after its commencement, stated that she was awoke in the morning by pain in her right eye, severe headache, and browache. She found that vision in this eye was almost completely lost, and complained of great intolerance of light, sensations of luminous flashes passing in front of the organ, and tenderness of the globe. A slight zone surrounded the cornea, and there was general fever. The disease was completely subdued, and vision restored by bleeding and mercury.

"The *treatment* of acute retinitis must be energetic in proportion to the activity of the symptoms, and the dangerous character of the disease; since, if the morbid action be not speedily arrested, such an amount of organic change may be produced as will certainly prove destructive to vision. Any considerable deposit of lymph, or even so much as to produce opacity of the texture of the retina, will be almost sure to be followed by this lamentable result."

We believe, however, that the prognosis is not so unfavourable, if active and judicious measures be commenced sufficiently early. In the Lancet for 1840-41, Vol. 2 (Essay 2nd), we find that Dr. Hocken states he "can prove that judicious measures are capable, not only of putting a stop to the symptoms, by curing their pathological origin, at an early period of their existence, but even of restoring vision after its complete loss,—provided that treatment be commenced before the functional loss has become permanent, or the effused lymph unsusceptible of the influence of mercury, by its becoming organized, and undergoing analogous changes." These assertions are illustrated and confirmed by a case.

Our author includes under "chronic retinitis," a description of several other forms of amaurosis, beside the varieties of the inflammation itself.

In fact, the section is similar to those general descriptions of amaurosis which we find in volumes where the author either considers and treats the subject as if it included but one disease, or where a general consideration precedes a description of the very different pathological states which are included under this generic term.

"Sometimes," says the author, "there is an involuntary rolling motion of the eyes, and this is more particularly observable in those cases in which the amaurosis is owing to some congenital disease; but it is not uncommon to witness it also, to a certain extent, even in persons who have been attacked during the adult period of existence. In some instances of the latter, I have noticed it in so slight a degree as scarcely to be perceived without a close inspection. This involuntary movement is commonly thought to be owing to the eyes being incessantly in search of light—a thirst for light as it has been fancifully termed—but as it is observed in cases of the most complete amaurosis, and is not always present even in congenital cases, I think it should be regarded rather as the result of some affection of the motor nerves, either in the brain, or in their passage through the orbit."

The author states that, although he has frequently witnessed the application of strychnia endermically, yet he has never been so fortunate as to observe any really useful results; "having, however, it must be stated, never employed it, except in cases that had resisted the use of ordinary remedies." Strychnia acts as a powerful stimulant to the nervous system, and should, therefore, never be employed during the progress of active congestions, or decided inflammation.

*Sympathetic or Functional Amaurosis.*—Very similar phenomena are sometimes witnessed in cases where there is not the least appearance of inflammatory action within the eye; in this event, says the author, the defective vision will probably be the result of some disorder, either of the optic nerve, or of the brain, or perhaps of the fifth nerve, and the disease is then termed sympathetic or functional amaurosis.

Usually, in amaurosis from affections of the brain, the patient complains of pain in the head, vertigo, and sometimes paralysis of other parts—as loss of sensation, or of power over the muscles of the face, or of the eyelids—denoted by a twist of the mouth, ptosis, or lagophthalmos. In children, amaurosis is often accompanied by the ordinary symptoms of hydrocephalus.

*Symptomatic Amaurosis.*—Amaurosis "is sometimes symptomatic of a disordered condition of some more remote organ, as the stomach, intestines, or the uterus. When there is no appearance of disease of the retina, and no sufficient indication of cerebral mischief to account for the defect of vision, we must try to discover whether or not some of the abdominal viscera be the seat of morbid action. Numerous visceral derangements are considered competent to the production of amaurosis; among these may be mentioned worms, indigestion, irregularity of the menstrual function, hysteria, and the like. That amaurosis is a frequent accompaniment of these morbid conditions there can be no doubt, but it is doubtful if the retina be ever influenced in this manner, except through the medium of the brain itself, the paralysis of the retina being to be regarded as resulting from disordered action of the brain. It may be a question how far the retina can be affected through the medium of the sympathetic nerve, but at present we have no sufficient data to reason upon. The proper mode of remedying this variety of amaurosis is by restoring the healthy function of the organ, to whose derangement the impairment of vision seems referrible; the means to be adopted are necessarily various, depending on peculiarities of age, sex, constitution, condition of the affected organ, and the like. It is not my intention, however, to enter upon the nature and treatment of the various morbid conditions which I have mentioned as giving rise to symptomatic amaurosis; I can only refer to the proper authorities for suitable information. Some very elaborate essays have been recently published in the journals, by Dr. Hocken, on symptomatic amaurosis, particularly from hysteria, to which the reader is referred. His volume on the same subject is also worthy of attention."

Our author imagines that, when vision is injured from affections of some of the branches of the fifth nerve—especially the frontal branch—sensibility to light only is lost (not vision), usually combined with a dilated pupil, or mydriasis—unless the nervous matter of the retina has suffered simultaneous concussion.

"The effects of blows or other injuries in the neighbourhood of the eye, are exceedingly various; sometimes the retina being paralysed, sometimes the iris, and at others the lens is rendered opaque; and vision will be impaired from any of these causes. It is very common for medical men, if they see a case of dilated pupil with imperfect vision, to set it down at once as a case of amaurosis, without enquiring how far the disturbance of vision is the result of paralysis of the iris, and whether the retina may be only secondarily affected. I repeat, therefore, that when there is paralysis of the retina accompanying dilated pupil, in this description of case, we are to regard the former as, in all probability, resulting from concussion of its texture, or of that of the optic nerve, or of the brain." 232.

We have little doubt that the symptoms often depend on mydriasis in these cases, and occasionally on injury of the eye, orbit, or brain, but not invariably on either. Dr. F. D. Walther, (Journ. der Chir. and Augenheilk, Oct. 1840,) from a careful review of the subject, arrived at the conclusion that injury of the fifth nerve had nothing to do with the production of amaurosis—1st, because division of the frontal branch is often effected without amaurosis; and 2nd, because it occurs when the nerve is intact. This reasoning however is most fallacious, inasmuch as those who have seen the greater number of such accidents do not contend that amaurosis occurs from every injury of the frontal branch, but when it is partially divided, or becomes involved and irritated in the subsequent cicatrix—hence the treatment recommended is to divide the nerve completely, and several cases are on record of its complete success. We have the testimony of so able an ophthalmic observer as the late Dr. Hunter to prove that true amaurosis occasionally follows an injury of one of the branches of the fifth nerve. He read a very curious and interesting case of "Temporary amaurosis of one eye following the extraction of a tooth," before the Med. Chir. Society of Edinburgh, April 7th, 1841.

Bearing in mind what we have already stated in reference to the function of the ophthalmic division of the fifth nerve—that filaments distributed with the retina probably supply that membrane with sensibility to the influence of light, and regulate the aperture of the pupil according to the quantity of light admitted into the interior of the eye—it seems most likely that the majority of cases, not depending on injury of the retina, optic nerve, or brain, are not cases of true amaurosis, but of insensibility to the irritating influence of light, and some imperfection and confusion of vision from mydriasis, or excessive dilatation of the pupil; whilst the few cases of true amaurosis are dependent on irritation of this branch, and not on its paralysis.

*Amaurosis from General Debility.*—Our author remarks, that among amaurotic patients there will sometimes be considerable difficulty in satisfying ourselves as to the kind of case we have to treat—whether it be one that has arisen from an enfeebled condition of the general system, or whether the amaurosis be the result of organic change in the structure of the retina, or of some portion of the brain or nervous system; and, as a consequence, we are at a loss to decide on the most appropriate treatment. In such cases, he says, it is better to trust to the internal exhibition of mercury, carefully avoiding the production of salivation, at the same time improving the general health by good nourishing diet, tonic medicines—as quinine, preparations of iron, and the mineral acids, country air, sea-bathing, and the like. Should there be any leucorrhœal or other debilitating discharge, this should be checked by every suitable means, and if suckling have been continued, it must be at once put a stop to. Though counter-irritation may be serviceable, yet it ought not to be of such a character as to produce any considerable discharge; hence, issues and setons are not to be commended, and sinapisms or stimulating linaments to the temple or nape of the neck are to be preferred.

To diagnose these cases, it is only necessary to investigate and analyse carefully *all the symptoms, both objective and subjective*, which are present. Bearing in mind that general debility, with an anæmic, or cachectic condition, often complicates amaurosis, dependent on disease going on within the eye, orbit, or brain, it becomes necessary to ascertain if any such exist, by attending to the peculiar symptoms of each. The phenomena of cerebral disease differ sufficiently from the symptoms of mere deficiency of the cerebral circulation to enable the attentive and well-informed practitioner to discriminate them successfully, and hence to adapt an appropriate treatment to each.

Passing over the sections on hemiopia and amaurotic cat's eye, we may remark that we fully agree with the author, that "glaucoma" is simply a form of internal inflammation, where the posterior chamber, instead of being perfectly transparent, exhibits an appearance of a dull green colour. "That these trifling deviations, depending upon accidental circumstances, should be thought worthy of distinct names, and indeed be described as distinct affections, is certainly not a little remarkable." Dissection has shown that, in these cases, the internal textures of the eye generally have been the seat of inflammation, which has led to various diseased appearances—the retina being probably the focus of morbid action. The retina has been found covered with a deposit of lymph, and otherwise altered; the vitreous humour, fluid and of a yellowish or greenish tint; the pigment of the choroid more or less absorbed, and its vessels varicose; and frequently the crystalline slightly discoloured or opaque, with sundry other changes characteristic of the existence of internal ophthalmia.

*Injuries of the Retina.*—The author endeavours to reprobate the idea, entertained by most persons, that the effects of injuries are in proportion to the presence or absence of resistence or preparation on the part of the patient, at the time of the accident.

"Most injuries of this sort are accidental, unexpected, and therefore no preparation can be made to ward off the means by which they are inflicted. If it be meant generally that a person who is expecting a blow, as in boxing, will be better prepared to ward it off, and that when an injury is received under such circumstances, the mischief inflicted on the eye will usually be less, so far may be readily granted; but if it be intended to mean that the same degree of violence applied to the eye, when the person is not expecting it, will produce more serious effects than when he is on the look out for the injury, then I do not perceive that the proposition is tenable." 241.

It seems to us that the author has taken a wrong, and somewhat ridiculous, view of the matter. We understand the common opinion that amaurosis is more likely to result from a slight injury if the eye be unprepared for violence—to mean that a blow may be inflicted on the eye whilst the lids are open, and the sufferer quite unconscious of his danger till he feels its effects. For instance, a person has been known to have received a slight blow on the cornea from a spent shot, and to have become amaurotic in consequence, whilst eagerly viewing some individuals shooting at pigeons; being himself situated at such a distance as to believe himself out of danger, and being struck before the lids had time to be closed, &c. &c. The rationale, also, of these facts appear to us perfectly clear, viz. that if the globe be struck whilst the lids are open, and the eye as prominent as usual, its effects are felt mainly in the nervous structures at the bottom of the organ; but if the lids are closed previously, the eyeball retracted, and the brow and cheek drawn more or less over the organ, in order to protect it—that is prepared for violence—the main effects are expended on the superficial structures, and the retina often escapes.

The term asthenopia is adopted from Dr. Mackenzie's Essay for "weakness of sight." The author thinks that the affection is unconnected with amaurosis, although it is commonly considered as a variety of it. If amaurosis includes every variety of defective vision, temporary or partial, dependent on nervous derangement, it is obvious that it can belong to no other genus. He considers that probably its essential nature is debility of the retina, rendering it incapable of sustaining any long-continued exertion. "Very likely, also, the nervous and muscular powers of the eye generally participate in the loss of energy." We fully concur with the author, that the benefits stated to have resulted from division of one or more of the muscles of the globe, "ought rather to be attributed to the repose which the eyes would receive afterwards, than to the operation itself."

We close the volume fully convinced that it is the production of an observant and intelligent mind. We have had to differ in opinion with the author on some important practical points—particularly on the apparent want of discrimination in the types of disease, and the selection of remedies, according to the peculiar features of the individual case; as well as an undue partiality for the application of the nitrate of silver to the conjunctiva. Some parts of the volume are too much extended, if they happen to partake of the author's favour, and others equally contracted. On the whole, however, the Oculist's Vade-mecum is an excellent practical digest of the present state of the art and science of ophthalmic medicine and surgery. The wood-cuts and coloured engravings are the worst part of the volume.

